# The Prevention of Ischemia-Reperfusion Injury in Elderly Rats after Lower Limb Tourniquet Use

**DOI:** 10.3390/antiox11101936

**Published:** 2022-09-28

**Authors:** Borja Herrero de la Parte, Javier Roa-Esparza, Iñigo Cearra, Inmaculada Ruiz Montesinos, Daniel Alonso-Alconada, Ana Alonso-Varona, Carmen Mar Medina, Sira Iturrizaga Correcher, Ignacio García-Alonso

**Affiliations:** 1 Department of Surgery and Radiology and Physical Medicine, Faculty of Medicine and Nursing, University of the Basque Country UPV/EHU, ES-48940 Leioa, Spain; 2 Interventional Radiology Research Group, Biocruces Bizkaia Health Research Institute, ES-48903 Barakaldo, Spain; 3 Department of Traumatology and Orthopedics, Basurto University Hospital, Osakidetza Basque Health Service, ES-48013 Bilbao, Spain; 4 Regenerative Therapies, Osteoarticular and Tendon Pathology Research Group, Biocruces Bizkaia Health Research Institute, ES-48903 Barakaldo, Spain; 5 Department of Gastrointestinal Surgery, Donostia University Hospital, Osakidetza Basque Health Service, ES-20014 Donostia, Spain; 6 Department of Cell Biology and Histology, Faculty of Medicine and Nursing, University of the Basque Country UPV/EHU, ES-48940 Leioa, Spain; 7 Department of Clinical Analyses, Galdakao-Usansolo Hospital, ES-48960 Galdakao, Spain

**Keywords:** ischemia-reperfusion injury, lower limb, elderly rats, prophylactic treatment, folinic acid, functional recovery

## Abstract

Background: Lower limb ischemia-reperfusion injury (IRI-LL) is a common major complication of orthopedic surgery, especially in elderly patients. It has previously been demonstrated that folinic acid (FA) reduced IRI-LL damage in 3–4-month-old rats. This current work analyses the effect of FA in the prevention of IRI-LL in elderly animals. Methods: Forty-two 18-month-old male WAG/RijHsd rats were subjected to 3 h of ischemia. Eighteen animals received FA (2.5 mg/kg, ip) 20 min before the end of the ischemia period, while the other half received the same volume of saline solution. The animals were sacrificed after 3 h, 24 h, and 14 days of reperfusion for biochemical (tissue damage markers and electrolytes), histopathological studies of the gastrocnemius muscle and the daily assessment of the limb function by the Rota Rod test, respectively. Results: The administration of FA prior to the end of the ischemia period reduced the increase in LDH and CK observed in non-treated animals by 30–40% (*p* < 0.0001). When the histological sections were analyzed, FA was found to have reduced the number of damaged muscle fibers per field by 20% (60 ± 17.1 vs. 80.7 ± 16.4, *p* < 0.0001). The functional test revealed that FA also led to an improvement in the muscle function, assessed by the length of time that the animals kept running on the rod, compared to untreated animals. Conclusions: The administration of FA, prior to the end of the ischemic period, decreases the damage induced by IRI-LL, also achieving a faster recovery of mobility.

## 1. Introduction

Nowadays, the use of tourniquets is a common practice in orthopedic and plastic surgeries all over the world. These compression devices prevent blood flow to the extremities in order to create a bloodless surgical field, with decreased perioperative blood loss, allowing surgical procedures to be performed with greater precision, safety and speed [1]. The first electronic tourniquet system was developed by Dr. James McEwen in the early 1980s [2]. Modern tourniquet systems are based on microcomputers and have built-in self-checking and self-calibration systems to ensure safety, prevent overpressure and provide precise control over the cuff pressure through electronic regulators and are equipped with pneumatic leak sensors and time-of-use counters [3]. These systems are classified by the U.S. Food and Drug Administration (FDA) as class I medical devices, which indicates that they present minimal harm to the patient and do not constitute a potential cause of injury. However, in spite of technological advances, localized secondary tissue damage due to cuff compression and injuries resulting from ischemia-reperfusion syndrome (IRI) continue to be reported [4].

The pneumatic pressure exerted by these devices generates a direct compression of the structures located under the tourniquet cuff, causing mechanical damage to the skin, muscles, nerves and vessels as a result of sagittal forces causing their compression and axial forces causing their stretching due to the uneven distribution of pressure under the cuff [4,5,6]. The pathophysiological mechanisms underlying IRI can be divided into two phases/stages: the ischemic state and the reperfusion state.

During ischemia, oxidative phosphorylation of ATP is inhibited. Anaerobic metabolism and electron transport chain dysfunction are induced in the mitochondria, leading to reduced ATP production and causing sodium–potassium pump (Na^+^/K^+^-ATPase) dysfunction, which increases intracellular Ca^2+^, H^+^ and Na^+^ levels. Ca^2+^ overload induces cell membrane destruction, hindering the mitochondrial function and triggering the activation of the inflammatory and apoptotic cascade [7]. When blood flow is restored, pathophysiological alterations are triggered by reactive oxygen species (ROS), the activation of the complement system and leukocyte activation [6,8,9]. The hypoxanthine accumulated in excess during ischemia is oxidized by xanthine oxidase (XO) during reperfusion, resulting in toxic levels of ROS. In turn, ROS stimulate leukocyte activation and chemotaxis, as well as the expression of cytokine genes and leukocyte adhesion molecules, through the activation of transcription factors such as nuclear factor-κB (NF-κB), also leading to direct cell injury [8,9]. The complement system and the formation of several proinflammatory mediators that alter vascular homeostasis (β 2 integrin, CD11b-CD18, tumor necrosis factor α, interleukin-1 and interleukin-6, among others) are also activated during reperfusion [6,10], jeopardizing local blood flow. In addition, activated leukocytes, when extravasated into the interstitial compartment, release toxic ROS, proteases and elastases, resulting in increased microvascular permeability, edema, thrombosis and parenchymal cell death [9,11].

From the clinical point of view, the refilling of the vessels and the reactive hyperemia cause an increase in the intra-compartmental pressure of the reperfused limb. Consequently, the circumference of the limb may increase by up to 50% on the first postoperative day, which may persist for 6 weeks after the procedure [12]. Additionally, lower limb IRI is often associated with weakness, stiffness, edema, dysesthesia and pain [13,14,15,16,17,18]. Such symptoms may be mistakenly attributed to surgical trauma or even to the patient’s lack of motivation [19].

Beyond this damage, which is restricted to the limb subjected to ischemia, systemic damage is of great importance due to the distribution of ROS and proinflammatory molecules throughout the organism that can lead to pulmonary, renal, cardiac, neurological and hepatic damage, among other effects, and even an increase in the surgical site infection [20,21,22,23,24]. 

A high percentage of total knee arthroplasties are performed on elderly patients in whom the muscle damage associated with the tourniquet is almost permanent and may increase the fall risk, as well as the patient’s loss of independence/autonomy [25,26]. According to the literature, all these morbidities are exacerbated when the tourniquet is applied to an elderly patient [27,28]. For example, a brief period of hypotension after tourniquet deflation, secondary to lactic and metabolic acidosis and hyperkalemia, may lead to myocardial depression and even cardiac arrest in elderly or debilitated patients after prolonged lower limb surgery [29]. This reminds us that special perioperative care is mandatory for elderly patients when they undergo extensive or prolonged surgeries [30,31]. In this sense, there is not much scientific evidence concerning the study of the preventive treatment of IRI in elderly patients [32,33] and even less on lower limb IRI (IRI-LL).

We previously published a model of IRI-LL in adult rats, validating a tension-controlled, self-made system designed to induce and maintain ischemia for 3 h, analyzing its effects at the biochemical, histopathological and functional levels. We even investigated the use of prophylactic treatment with folinic acid (FA) to prevent IRI-LL-associated damage. 

Folinic acid is the active analog of folic acid in the form of a salt, which is available for parenteral administration. This vitamin is an essential factor in different biological pathways, such as DNA and RNA synthesis, cell replication, intracellular signaling and gene methylation [34].

In this context, folate deficiency is associated with several disorders. For example, it affects hematopoiesis, leading to megaloblastic anemia [35]. During fetal development, a high number of mitoses occur; thus, folate deficiency can lead to neural tube defects [36]. The therapeutic effects of folates in improving fertility, certain depressions, or even reducing the risk of age-related macular degeneration have been demonstrated [37,38,39,40].

In the context of oxidative stress related to IRI, FA is also known to play a protective role by reducing homocysteine levels (related to free radical formation and the dysregulation of antioxidant mechanisms), as well as by its ability to competitively inhibit xanthine oxidase (XO) due to its structural analogy with the substrates of this enzyme. If these processes do not progress properly, reactive oxygen species (ROS) can be produced and lead to DNA damage [41,42,43].

FA has also been investigated in models of IRI in cardiac muscle. During the ischemia-reperfusion process in this tissue, a deficient expression of endothelial nitric oxide synthase (eNOS) is generated, which requires its cofactor, tetrahydrobiopterin (BH_4_), to carry out its function. FA is able to stabilize the amount of BH_4_ from the oxidized and inactive form BH_2_, ultimately limiting vascular dysfunction and ischemia-reperfusion damage [41,44,45]. In addition to this process, it also involves the scavenger activity of reactive oxygen species [46]. Similar findings were reported by several studies on the mesenteric territory, retina and hepatic liver conducted by our research group [43,47,48,49,50]. For example, Portugal et al. [48], in a model of hepatic ischemia associated with liver resection, demonstrated a reduction in oxidative stress related to ischemia and an increase in the percentage of hepatic regeneration in those who received 2.5 mg/kg of FA.

In this context, the aim of this work was to characterize the damage induced by a 3 h ischemia period and to assess the potential of FA to prevent such damage in elderly rats.

## 2. Materials and Methods

This project was approved by the Institutional Review Board of the University of the Basque Country (ref. M20/2020/303) and was conducted in compliance with the current national and European Union regulations for the protection of animals used for experimental and other scientific purposes.

Forty-two elderly WAG/RijHsd male rats aged around 18 months-old, with a mean weight of 338 ± 24 g, were randomly distributed into 6 experimental groups (6 animals for each group) and an additional control group (another 6 healthy animals not subjected to any procedure). Groups 1 and 2 (3 h of reperfusion) were used for biochemical studies. Groups 3 and 4 (24 h of reperfusion) provided the samples for the pathological analysis. Finally, groups 5 and 6 (14 days of reperfusion) were used for both biochemical and functional studies. The animals in groups 2, 4 and 6 were treated with folinic acid, while the animals in groups 1, 3 and 5 received the equivalent volume of the vehicle (saline). 

### 2.1. Ischemia Induction and Maintenance

A self-designed device was used for the induction of ischemia and its maintenance for 3 h [51]. In order to properly position the animal in the device, the animal was sedated with an intraperitoneal injection of diazepam (15 mg/kg). Ten minutes later, once the animal was completely sedated, ketamine (80 mg/kg, ip) and medetomidine (0.5 mg/kg, ip) were administered. The animal was placed in the supine position and, after draining the blood contained in the blood vessels of the limb using an Esmarch bandage, a pressure-controlled tourniquet (1 kg) was positioned at the base of the hip joint.

Then, the rubber band was carefully removed and the achievement of the correct degree of ischemia was verified by laser Doppler (moorVMS-LDF1, Moor Instruments Ltd., Devon, UK) in the territory of the pedicle artery, and the visualization of the clinical symptoms of ischemia (pallor and absence of capillary refill) was carried out. The foot was held at zenith traction using one of the auxiliary arms of our ischemia device. Seventy to ninety minutes after the onset of ischemia, another dose of diazepam (7.5 mg/kg) was administered.

According to the experimental groups described above, FA (2.5 mg/kg; Folidan^®^ 50, Almirall Prodesfarma, Barcelona, Spain) or saline was intraperitoneally administered 20 min before releasing the tourniquet and restoring the blood flow to the limb. The adequate restoration of the blood flow to the limb was verified by laser Doppler and the appearance of clinical signs (reactive hyperemia and adequate capillary refill) was observed. Post-ischemia recovery was accomplished using a heat source at 22–24 °C, and buprenorphine (0.05 mg/kg sc) was administered to minimize post-procedural pain. The animals were allowed to progress for 3 h, 24 h or 14 days, according to each experimental group, in order to evaluate the results at the biochemical, histopathological and/or functional levels.

### 2.2. Biochemical Analyses

Biochemical changes were quantified for 3 h or 14 days after ischemia (the reperfusion period). Blood samples were collected from the abdominal cava vein. The animals were anaesthetized by 1.5% isoflurane and, after exposure of the cava vein, blood samples were collected with 20 G needle and immediately transferred to tubes for serum separation, without additives and with separating gel (BD Vacutainer^®^). The serum was obtained by centrifugation at 3000 rpm for 10 min. Electrolytes (Na^+^, K^+^ y Cl^−^), renal function markers (urea and creatinine), liver markers (aspartate aminotransferase (AST) and alanine aminotransferase (ALT)), muscle damage markers (creatinine kinase (CK) and lactate dehydrogenase (LDH)) and alkaline phosphatase (AP) were quantified using Cobas^®^ 8000 modular analyzer (Roche Diagnostics, GMBH, Mannheim, Germany).

### 2.3. Pathological Studies

Histological changes due to ischemia-reperfusion injury were analyzed 24 h after the ischemia period. The perimeter, the weight and the number of damaged fibers were quantified. Under 1.5% isoflurane anesthesia, the rat was repositioned in the ischemia device, and the limb was fastened with the auxiliary arm. A 1/0 silk ligature was passed around the limb, and the perimeters of both limbs were obtained at the hip joint by measuring the length of the ligature with a ruler. Then, both the ischemic and contralateral healthy gastrocnemius muscles were carefully excised, the weights were recorded and the muscles were immersed in 4% paraformaldehyde. After 24 h, the specimens were embedded in paraffin, and 5 µm histological sections were obtained.

For the microscopical examination, slices were stained with hematoxylin/eosin, and five photographs were obtained from each animal at a 66× magnification. The number of polymorphonuclear neutrophil (PMN) cells and of total fibers and damaged fibers in each photograph were quantified. Fibers were considered damaged if they presented with broken or blurred edges, a non-uniform texture and color, holes in the cytoplasm and/or separated or detached cell nuclei. On the other hand, fibers were considered intact if they presented with well-defined edges, with the same texture and uniformity along the cell membrane, without holes or breaks, and with a correct visualization of the pericellular nuclei and satellite cells. 

This histological assessment of the sections was performed blind to the principal investigator and analyzed by another member of the team. The objective analysis was thus ensured, since it was not known whether a sample was obtained from the ischemic or contralateral limb, or whether or not it had received the experimental treatment with FA.

### 2.4. Functional Study: Rotarod Test

The functional recovery after IRI-LL was assessed using a rotarod apparatus (Panlab Harvard Apparatus, Barcelona, Spain). The rotation speed and acceleration, as well as the time for acclimatization to the rod, were the same as those indicated in previous works of our group [51,52]. The latency time to fall, as well as the speed of the rod at the time of the fall, were automatically recorded using the SeDaCom v2.0.03 software (Panlab Harvard Apparatus, Barcelona, Spain). As previously described, the animals were subjected to prior training to acclimate the rats to the rod.

### 2.5. Euthanasia

The euthanasia of the animals was carried out in accordance with the current legislation, ensuring animal welfare during the whole procedure and minimizing the stress incurred during the process. In the case of those animals for which blood collection was performed (groups 1, 2, 5, 6 and the control), the blood withdrawal for further analysis necessitated the death of the animal by exsanguination. On the other hand, in groups 3 and 4, from whom blood samples were not collected and only muscle samples were taken, euthanasia was carried out by the intraperitoneal injection of sodium pentothal (90 mg/kg).

### 2.6. Statistical Studies

The statistical analysis was performed using the Prism v8.2.1 statistical software (GraphPad Software, San Diego, CA, USA). The results were verified for normality by the Kolmogorov–Smirnov test and expressed as the mean and standard deviation. Comparisons between the two experimental groups (treated and untreated) were performed using a two-tailed *t*-test for unpaired samples. A two-tailed t-test for paired samples was also performed when comparing both limbs of the same animal (healthy and ischemic limbs) or in the functional study evaluating the evolution of each animal over 14 days. For the comparison between 3 or more experimental groups, an analysis of variance (ANOVA) was performed with the Newman–Keuls test for multiple comparisons between groups. A confidence level of 95% was established for all cases.

## 3. Results

Firstly, based on a qualitative assessment, all the animals subjected to ischemia using our self-made device successfully completed the 3 h ischemia period under the pressure-controlled tourniquet, with no intra- or post-procedural complications reported. There were no deaths as a consequence of the subsequent reperfusion, and only the animals in the untreated group showed impaired mobility in the immediate postoperative period (up to 24 h). No weight loss greater than 15% was observed in the animals analyzed 14 days after ischemia.

### 3.1. Biochemical Analyses

**Figure 1:**
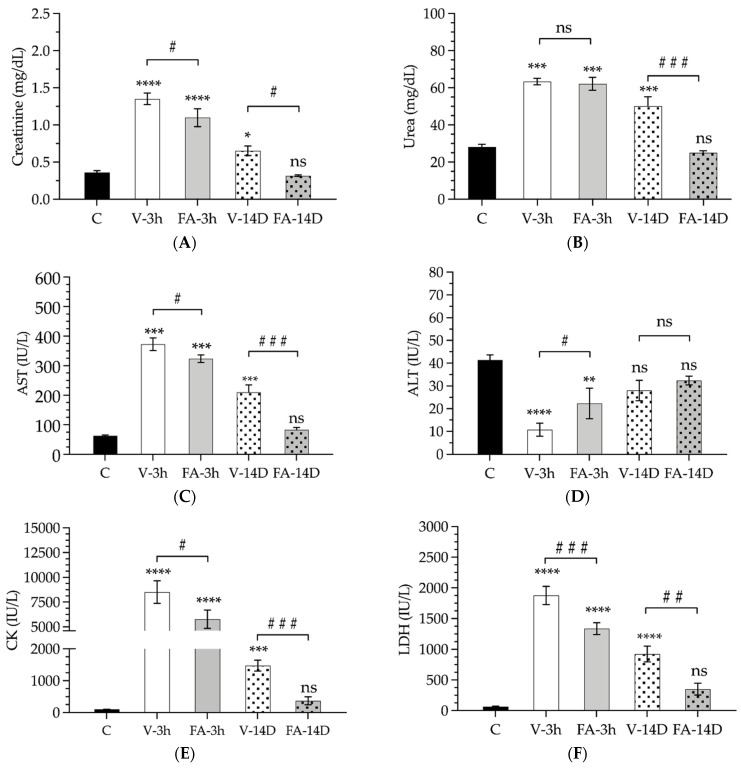
Enzyme and electrolyte levels detected in the serum samples collected from the control animals (C, black bar) or animals subjected to ischemia after a period of 3 h or 14 days of reperfusion and treated with folinic acid (FA-3h, smooth gray bars; FA-14D, smooth gray bars) or treated with saline (V-3h, smooth white bars; V-14D, smooth white bars): creatinine (**A**), urea (**B**), aspartate transaminase (AST) (**C**), alanine transaminase (ALT) (**D**), creatine kinase (CK) (**E**), lactate dehydrogenase (LDH) (**F**). Asterisks indicate statistically significant differences compared to the control. Pads indicate statistically significant differences between the untreated animals (white bars, vehicle) and those treated with folinic acid (grey bars, FA) after both 3 h (smooth pattern) and 14 days (dotted pattern). Data are represented as the mean and standard deviation. Statistical significance is accepted with a 95% confidence level (#, *: *p* < 0.05; ##, **: *p* < 0.01; ###, ***: *p* < 0.001; ****: *p* < 0.0001; ns: *p* > 0.05).

The serum samples obtained after 3 h or 14 days of reperfusion were analyzed for renal markers (creatinine and urea), transaminases (AST and ALT), rhabdomyolysis markers (CK and LDH) and electrolytes (Na^+^, Cl^−^, K^+^) (Figure 1A–F and Table 1).

Both the creatinine and urea levels showed a similar pattern. After a 3 h reperfusion period, IRI-LL induced a significant elevation in both creatinine (1.4 ± 0.22 vs. 0.36 ± 0.06 mg/dL; *p* < 0.0001) and urea (63.4 ± 5.1 vs. 28.1 ± 3.8 mg/dL; *p* < 0.001). FA treatment did not induce any significant change. On day 14th, both creatinine and urea levels were found to have decreased significantly but only reached normal values with the FA treatment (*p* < 0.0001) (Table 1, Figure 1).

Following 3 h of reperfusion, the AST levels showed a similar trend to that described above. Compared to saline administration, the FA treatment exerted no substantial effect (*p* > 0.05) (Table 1, Figure 1C). Fourteen days after being subjected to ischemia, non-treated animals still showed elevated AST levels (210 ± 61.1 IU/L; *p* < 0.001); however, FA-treated animals reached normal values (83.2 ± 16.3 IU/L; *p* > 0.05). The ALT enzyme showed the opposite behavior (Table 1 and Figure 1D). After 3 h of reperfusion, its values decreased 4-fold compared to the control (41.4 ± 5.9 vs. 10.7 ± 8.1 IU/L; *p* < 0.0001). The preventive treatment with FA reduced this decrease by slightly more than 50% (22.3 ± 16.4 IU/L; *p* < 0.001). Finally, fourteen days after ischemia, both the non-treated and treated animals recovered normal ALT values.

The parameters that provided the best evidence of muscle damage, CK and LDH, showed a sudden elevation after 3 h of reperfusion (Table 1, Figure 1). The CK levels were 80 times higher than in the control group, reaching up to 8512 ± 3199 IU/L. This increase was reduced by more than 30% when the FA treatment was administered (5777 ± 2255 IU/L; *p* < 0.05). Fourteen days later, the animals that received saline still had extraordinarily high levels of CK (1476 ± 415.7; *p* < 0.001). On the other hand, though CK in the FA-treated animals was still slightly increased, the difference did not reach statistical significance.

LDH showed a rather similar behavior. Serum values increased by up to 30-fold after 3 h of reperfusion (1878 ± 388. 8 IU/L) and by more than 10-fold after 14 days (922 ± 314.1 IU/L). FA succeeded in reducing this increase by 30% after 3 h (1336 ± 231.4 IU/L) and by 60% after 14 days (350.8 ± 212.9 IU/L). 

Finally, the fluctuations in the electrolyte levels (Na^+^, Cl^−^ and K^+^) were not significant from a quantitative point of view, but in the case of Na^+^ and Cl^−^, they were statistically significant (Table 1).

### 3.2. Gastrocnemius Muscle Analyses: Macroscopic and Microscopic

**Figure 2:**
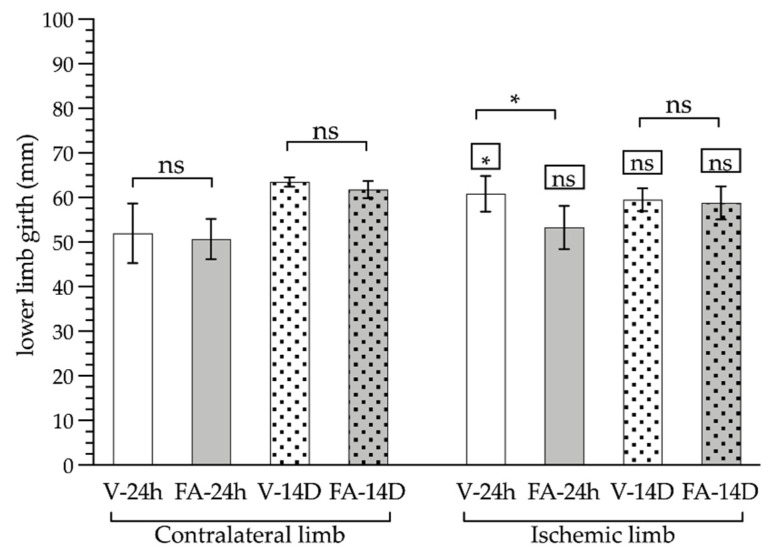
Measurements of the perimeter (in millimeters) of the limb subjected to ischemia (right limb) and the healthy contralateral limb (not subjected to ischemia, left limb), after a period of 24 h (smooth bars) or 14 days (dotted bars) of reperfusion and treatment with folinic acid (FA, gray bars) or saline (V, white bars). The statistical significance levels shown in the squares above the bars show the results of the statistical comparison between the ischemic limbs and the healthy contralateral limbs in the same experimental group (paired analysis). The significance levels shown in the square brackets refer to the comparison between the groups indicated (*: *p* < 0.05; ns: *p* > 0.05).

Following ischemia, the perimeter of the ischemic limb (Figure 2), measured at the hip joint, was increased by 15% (60.83 ± 4.02 vs. 52 ± 6.69 mm; *p* < 0.05). FA successfully prevented the increase in the limb circumference (53.29 ± 4.85 mm). Compared to the untreated animals, FA reduced the ischemic limb circumference by 14%. 

Fourteen days later, no significant effect of the FA treatment on the ischemic limbs compared to non-treated animals was identified (58.8 ± 3.7 vs. 59.5 ± 22.58 mm, respectively). It should be noted that there was a considerable increase in the perimeters of both the FA-treated and untreated contralateral limbs compared to the ischemic limbs (62.73 ± 1.67 vs. 59.18 ± 2.99 mm; *p* < 0.01) (Figure 2).

**Figure 3:**
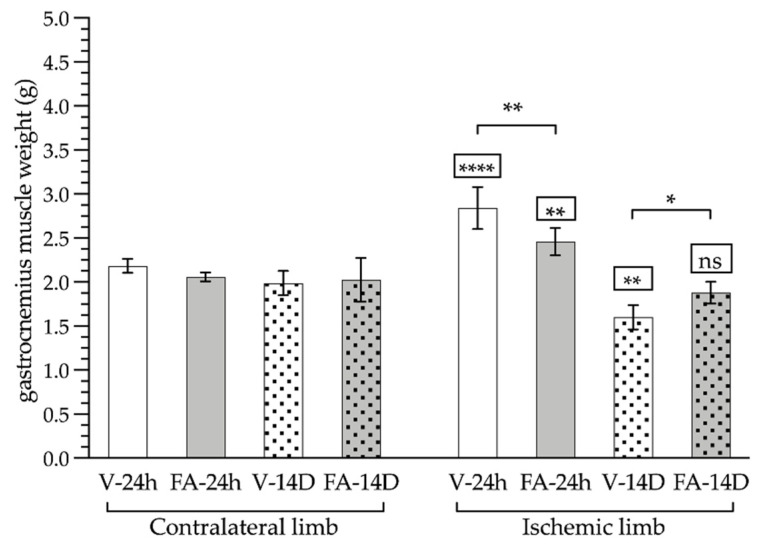
Weight (grams) of the ischemic limb (right limb) and the contralateral healthy limb (not subjected to ischemia, left limb) after a 24 h (smooth bars) or 14 day reperfusion period (dotted bars) and treatment with folinic acid (FA, gray bars) or saline (vehicle, white bars). The statistical significance levels shown in the squares above the bars show the results of the statistical comparison between the right and the left limbs in the same experimental group (paired analysis). The significance levels shown in the square brackets refer to the comparison between the groups indicated (*: *p* < 0.05; **: *p* < 0.01; ****: *p* < 0.0001; ns: *p* > 0.05).

The weight of the gastrocnemius muscle of the limb not subjected to ischemia remained constant despite treatment and was not modified throughout the days of the experiment. In contrast, in the ischemic limb, 24 h after ischemia, the weight of the gastrocnemius muscle in the untreated animals was increased by 30% compared to the contralateral limb (2.84 ± 0.24 vs. 2.18 ± 0.08 g; *p* < 0.0001). However, in those animals that received FA, this increase was reduced to just 20% (2.46 ± 0.15 vs. 2.06 ± 0.05 g; *p* < 0.0001). On day 14, while the untreated animals still showed a significant reduction in the gastrocnemius weight (1.60 ± 0.14 g; *p* < 0.05), in the FA treated animals, the muscle recovered its normal weight (1.88 ± 1.24 vs. 2.02 ± 0.25 g; *p* > 0.05) (Figure 3).

**Figure 4:**
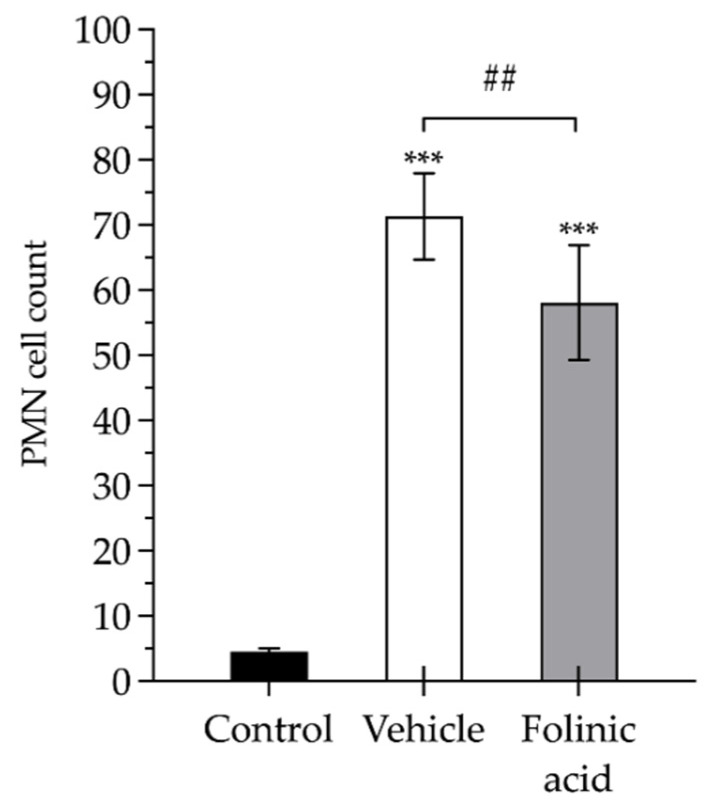
Polymorphonuclear neutrophil (PMN) inflammatory cell count in the gastrocnemius muscles of healthy control animals (control, black bars) and animals subject to 3 h of ischemia and 24 h of reperfusion and treated with saline (vehicle, white bars) or treated with folinic acid (gray bars). The pads show the statistically significant differences between the groups indicated by the upper box (## *p* < 0.01), and the asterisks show the statistically significant differences compared to the control (*** *p* < 0.001).

Finally, concerning the pathological analysis, it was found that, compared to healthy muscles (control), the muscles obtained from the animals subjected to ischemia showed a significant increase in the inflammatory infiltrate of PMNs (4.6 ± 2.5 vs. 71 ± 6.6 cells, respectively; *p* < 0.001). The FA administration significantly reduced the PMN count by 20% compared to the untreated animals (58 ± 8.8 cells; *p* < 0.01) (Figure 4). In terms of the PMN density, the cell counts translated into PMN densities of 0.38 × 10^4^ PMN/mm^2^, 5.94 × 10^4^ PMN/mm^2^ and 4.84 × 10^4^ PMN/mm^2^, respectively.

**Figure 5:**
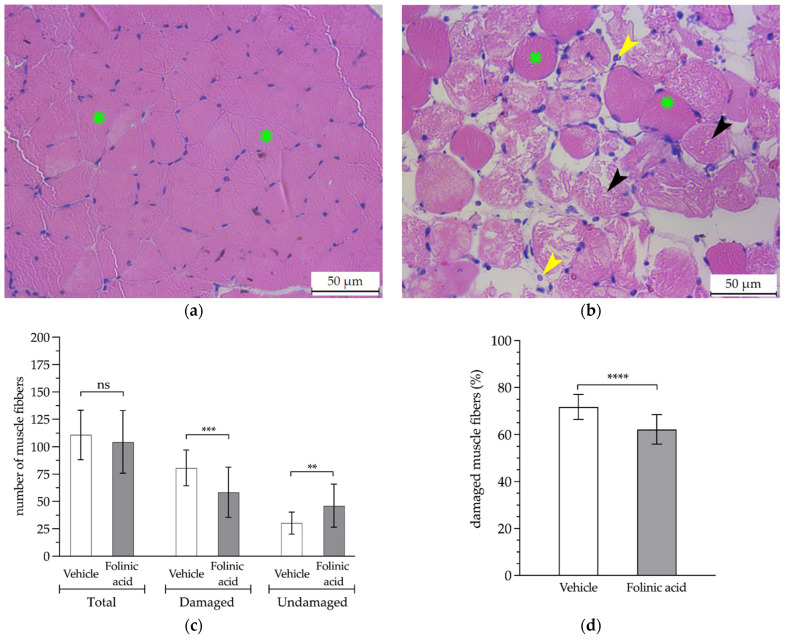
Microscopic analysis of the damage to the gastrocnemius muscle. Histological sections of the gastrocnemius muscle of both the contralateral healthy (**a**) and non-treated ischemic lower limbs (**b**), showing the non-damaged muscle fibers (green asterisk), the damaged muscle fibers due to the ischemia process (black arrowhead), and the inflammatory infiltrate of the polymorphonuclear cells (yellow arrowhead). The count of the total number of muscle fibers, damaged and undamaged (**c**), and the percentage of damaged fibers relative to the total number of fibers (**d**) for each histological section analyzed at a 66× magnification (**: *p* < 0.01; ***: *p* < 0.001; ****: *p* < 0.0001; ns: *p* > 0.05).

The microscopic observation of the gastrocnemius muscle slices showed that the ischemic limbs of non-treated animals exhibited a higher percentage of fiber damage compared to the FA-treated animals (80.7 ± 16.4 vs. 62.2 ± 6.2%, respectively; *p* < 0.001). The total fiber count did not show significant changes when comparing the vehicle and the FA administration (110 ± 22.5 vs. 104.4 ± 28.6 fibers; *p* > 0.05) (Figure 5).

### 3.3. Functional Study: Rotarod Test

**Figure 6:**
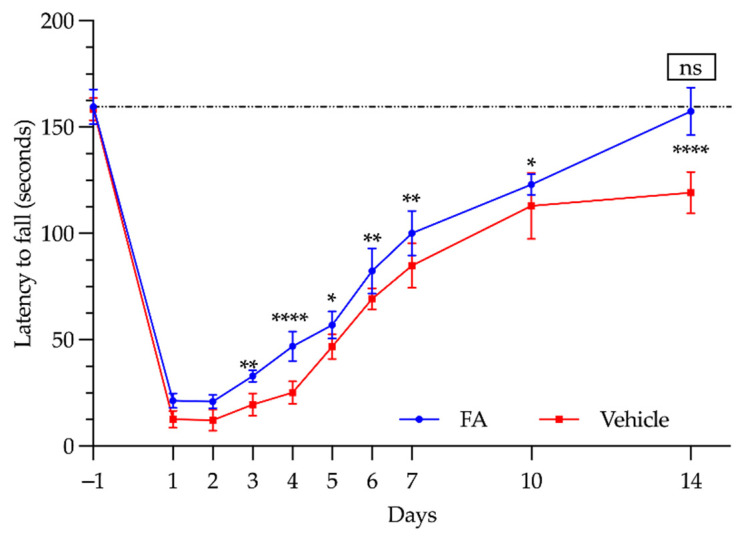
Rotarod test. The latency time to fall (seconds, s) of the animals treated with saline (vehicle, red line) and of the animals treated with folinic acid (FA, blue line). The box indicates the statistical significance in relation to the time obtained before ischemia was performed (*: *p* < 0.05;**: *p* < 0.01; ****: *p* < 0.0001; ns: *p* > 0.05).

Physical tests (the rotarod test) were performed over a 14-day period to evaluate the impact of IRI-LL on the functional capacity of the animals. As can be seen in Figure 6 and Table 2, both the vehicle and FA-treated animals showed a similar recovery trend.

In the 2 days following ischemia, no differences due to the prophylactic treatment were observed. The animals endured a similar time on the rotarod roller (ranging from 12 to 21 s). By the third day, significant differences could be observed in those animals treated with FA, with the greatest difference between the two groups reached on the fourth day, when the FA-treated animals endured up to 86% more time than those that were not treated (47 ± 7 vs. 25.2 ± 5.3 s; *p* < 0.0001).

It should be noted that, from the 10th day, the untreated animals showed a plateau in terms of the time that they were able to remain on the rod (113 ± 15.6 s), while the FA-treated animals showed a marked improvement until the end of the experiment on day 14 (157.4 ± 11.24 s), reaching similar values to those recorded prior to the ischemia induction (159.6 ± 8.3 s; *p* > 0.05).

## 4. Discussion

The statistical office of the European Union (Eurostat) reports that the population aged 65 years or more among the 27 member states of the European Union (EU-27) will rise by 40% by 2050, with a 56% increase in the population aged between 75 and 84 years [53].

However, the increase in the disease-free life expectancy has not shown the same linear increase as the life expectancy, mainly due to an increase in the incidence of chronic diseases such as cardiovascular, renal, metabolic or neurodegenerative diseases. For example, Spain has one of the highest life expectancies in the world (84 years for women and 80 years for men); however, the disease-free life expectancy decreased between 2006 and 2017 from 3.92 years to 3.45 years [54].

In this context, it is logical to assume that the number of orthopedic surgeries performed on elderly patients will also increase, since muscle weakness, pain, balance disorders, osteoarthritis or reduced mobility associated with aging increases the risk of falling, which is the main cause of the need for orthopedic surgery in the elderly [55,56,57].

As mentioned above, orthopedic limb surgery is usually performed using a tourniquet in order to achieve a blood-free surgical field. Its use is associated with both tissue damage due to the compression of the tourniquet itself, and with IRI, and it leads to an increase in hospitalization and the recovery time [58,59]. In debilitated and older patients, after the tourniquet release, a short period of hypotension, associated with acidosis and hypercalcemia, can lead to myocardial depression or cardiac arrest, and even generate states of cerebral hypoperfusion [4]. It is therefore necessary to evaluate the efficacy of prophylactic treatments that, in the context of limb surgery using a tourniquet, allow for the minimization or even avoidance of both local and systemic damage associated with IRI-LL.

In this work, we used the same device and the same rat strain as those used in previous works published by our research group, as well as the same battery of analyses (biochemical, pathological and functional) [51,52]. We selected the rat as the experimental model because of the similarities between the rat’s anatomy and that of humans, comprising the same muscle groups. Furthermore, the rat has the same biochemical processes as the human being and the same enzymes, which are used for biochemical analysis [60]. Therefore, the response of the rat musculature to ischemia-reperfusion-related events can be compared to the damage caused in humans. These studies, carried out on young adults (3–4 months), confirmed the efficacy of our device in inducing and maintaining controlled ischemia [51], as well as the usefulness of FA as a prophylactic treatment for reducing IRI-LL [52].

Comparing our study with that published by Cearra et al. [52], we can see that, in older animals, the AST values were elevated after 3 h of reperfusion, almost to the same extent as those of younger animals (372.8 ± 393.2 IU/L, respectively). However, the response of the ALT is diametrically opposed between older individuals and in young adults and also differs from that reflected in other studies on hepatic IRI [33,61,62,63]. In our work, aged rats suffered a sudden decrease in the ALT levels, reaching up to 75% with respect to the control values after 3 h of reperfusion. This phenomenon may be explained by a decrease in the production or release of ALT from the liver, or by a loss of functional liver mass or its enzymatic activity associated with reperfusion injury [64], although, in our case, this possibility could not be confirmed. Fourteen days later, the ALT levels returned to normal, while the AST levels, although decreased, remained elevated compared to the control.

When FA was administered prophylactically, the AST values improved slightly after 3 h of reperfusion but still remained different from the baseline figures. After 14 days, FA did achieve the complete normalization of the AST values. Huang et al. reported findings similar to ours when they administered a prophylactic treatment with hydrogen sulfide (H_2_S), which was shown to improve the tolerance to ischemia-reperfusion in both intestinal and other tissues. While, in young rats, a significant decrease in the serum AST and ALT values was observed, in older rats this improvement was very slight (*p* > 0.05) [63]. However, Hide et al. failed to reverse the liver IRI damage assessed by elevated AST and ALT levels in elderly rats, although they did demonstrate an improvement in the hepatocellular damage from the pathological point of view [33]. 

By analyzing muscle damage measured by the CK and LDH serum levels, we observed that, in aged animals, the serum values after 3 h of reperfusion were 10 and 30% higher, respectively, than those in 3–4-month-old rats. After 14 days, the CK levels were also higher in the aged rats (2.6-fold), while the mean LDH levels were 0.7-fold lower [52]. The FA treatment was slightly more effective in terms of both CK and LDH at 3 h and 14 days in elderly rats. This could be explained by the fact that the initial elevation was also higher, mainly due to the decline in the endogenous antioxidant defense in the elderly subjects [65].

Urea showed a similar trend in both adult and elderly animals after 3 h of reperfusion, but the FA administration did not lead the same response. While, in adult rats, FA was able to reduce the urea levels, in aged rats no effect was observed. Cearra et al. demonstrated that both the urea and creatinine elevations can be reverted endogenously by adult animals, with no additional effect of the FA administration [52]. However, in our work, we noticed that the old rats were unable to reverse the elevated levels of both urea and creatinine after 14 days on their own, and the elevated values could only be restored by administering FA. Accordingly, Jankauskas et al., in a model of renal IRI, found that both adult and old rats suffered a similar increase in the urea levels after reperfusion, but only 3–4-month-old rats benefited from ischemic preconditioning (IPreC) maneuvers in an attempt to diminish the increase in the urea levels after reperfusion [66]. Finally, we would like to note that, due to the slight fluctuations in the electrolyte levels found not only in this study but also in our previous experiments [51,52], these parameters could be omitted in future trials, as they do not provide any relevant information.

The pathological findings, after 3 h of ischemia and 24 h of reperfusion, showed that 80.7 ± 16.4% of the fibers were damaged. These figures correlate with those reported by McCormack et al. [67], with 60% of the fibers damaged after 2 h of ischemia, and by Deune et al., with 80% of the fibers damaged after 4 h of ischemia [68]. FA pre-treatment reduced the number of damaged muscle fibers in each field by 20% (64 ± 17.1 vs. 80.7 ± 16.4; *p* < 0.001), resulting in a reduction in damage similar to that achieved in younger rats with the same treatment (52.1 ± 20.6% vs. 65.5 ± 14.1%; *p* < 0.001) [52].

We also observed a reduction in the inflammatory cell count (PMN) after treatment with FA. This could indicate an anti-inflammatory or preventive effect on the migration of inflammatory cells to the ischemic region. These findings correlate with those previously published by different research groups. Huang et al. and Tommy et al. demonstrated that FA has anti-inflammatory properties in a traumatic brain injury model and also mitigates the anti-inflammatory response triggered by hypoxia [69,70]. In these studies, the anti-inflammatory effect is mediated by the inhibition of the phosphatidyl inositol-3 kinase/protein kinase B (PI3K/Akt) and hypoxia-inducible factor 1-alpha (HIF-1α) signaling pathways [69] and by the inhibition of the serum levels of homocysteine, tumor necrosis factor-alpha (TNF-α), interleukin 10 (IL-10) and the high-mobility group box 1 protein (HMGB1) expression [70]. Feng et al. also suggested that FA might act on activated macrophages [71]. It is initiated by the suppression of mitogen-activated protein kinase (MAPKs) and activation of NF-κB, leading to the inhibition of nitric oxide (NO), TNF-α and interleukin-1 beta (IL-1β) formations.

Concerning the macroscopic parameters of the hindlimb, Bonheur et al. [72], following 3 h of ischemia and 24 h of reperfusion, described a 22% weight gain, which is higher than the values we reported in this study (16%; 2.5 ± 0.38 g vs. 2.16 ± 0.12 g).

Furthermore, it was found that, similar to the results observed in younger rats [52], there was no significant difference in the gastrocnemius muscle weight when comparing the FA and saline treatment groups. Nevertheless, it was observed that FA significantly reduced the perimeter of the ischemic limb compared to the untreated group (11%).

Finally, from a clinical point of view, the functional outcomes are probably the most relevant. Several tests for their evaluation are described in the literature: the measuring of the isometric contractile force of the tendon of the muscle of interest [73], electromyography studies of the involved muscles [74], the use neuromuscular score charts [75,76], or even by the use a forced movement test, such as the rotarod test [77]. Orban et al. [76], for example, used the ASIA scale to assess the quadriceps of patients undergoing orthopedic surgery with a tourniquet. They found that there were no statistically significant differences in the muscle function between the control group and those patients treated with acetylcysteine and subjected to ischemic preconditioning.

In our study, we used the rotarod test, which has been widely used in models of cerebral stroke and neurodegenerative diseases or in the study of the effects of drugs on the central nervous system. However, we did not find any published work concerning its use to assess the locomotor function after IRI-LL. Chan et al. employed this test in a model of cerebral ischemia [77]. They observed that this test was useful for assessing the improvement in motor functions after remote ischemic preconditioning. Three days after cerebral ischemia, the animals undergoing preconditioning remained twice as long in the rotarod test as the control group. The same timing was also observed in our IRI-LL experimental model, as we were also able to demonstrate a significant improvement in the FA-treated animals after the third day.

## 5. Conclusions

In conclusion, in this work, we were able to demonstrate that prophylactic treatment with folinic acid prior to reperfusion led to a notable improvement in those animals subjected to ischemia through the use of a tension-controlled tourniquet from the functional, biochemical and pathological points of view. Further studies are required to determine the specific signaling pathways involved in the protective effect of FA on IRI-LL.

Our promising results contribute to and support the design and execution of clinical trials to test the use of folinic acid in clinical practice, as it is a safe drug with no toxicity, and could fill the current gap in knowledge regarding the management of elderly patients undergoing scheduled ischemia for lower and/or upper limb surgery.

## Figures and Tables

**Table 1:** Biochemical parameters quantified using the serum samples from the control animals and animals treated with saline (vehicle) or folinic acid (FA) after 3 h or 14 days. Aspartate transaminase (AST), alanine transaminase (ALT), creatine kinase (CK), lactate dehydrogenase (LDH).

Group	Control	Vehicle 3 h	FA 3 h	Vehicle 14 Days	FA 14 Days
Creatinine (mg/dL)	0.36 ± 0.06	1.4 ± 0.22 ****	1.1 ± 0.29 ****	0.65 ± 0.15 *	0.32 ± 0.02 ns
Urea (mg/dL)	28.1 ± 3.8	63.4 ± 5.1 ***	62.2 ± 8.6 ***	50.2 ± 8.5 ***	25 ± 2.6 ns
AST (IU/L)	62.6 ± 8.6	372.8 ± 48.3 ***	324 ± 28.6 ***	210 ± 61.1 ***	83.2 ± 16.3 ns
ALT (IU/L)	41.4 ± 5.9	10.8 ± 8.1 ****	22.3 ± 16.4 **	28 ± 10.9 ns	32.4 ± 4.4 ns
CK (IU/L)	101.5 ± 10.7	8512 ± 3199 ****	5777 ± 2255 ****	1476 ± 415.7 ***	371.8 ± 275.9 ns
LDH (IU/L)	66 ± 26.6	1878 ± 388.8 ****	1336 ± 231.4 ****	922 ± 314.1 ****	351 ± 213 ns
Na^+^ (mEq/L)	146.7 ± 0.9	138.1 ± 3.3 ***	137.3 ± 2.3 ***	139.8 ± 1.9 ***	141.6 ± 1.1 ***
K^+^ (mEq/L)	3.99 ± 0.45	4.4 ± 0.57 ns	4.3 ± 0.39 ns	4.38 ± 0.43 ns	4.24 ± 0.19 ns
Cl^−^ (mEq/L)	103.7 ± 1.5	88.6 ± 1.9 ***	90.7 ± 2.8 ***	94.7 ± 1.6 ***	101.4 ± 1.1 ns

Mg/dL: milligrams per deciliter; IU/L: international units per liter; mEq/L: milliequivalents per liter. All data are reported as the mean and standard deviation (SD). Asterisks indicate statistically significant differences compared to the control. Statistical significance is accepted with a 95% confidence level (*: *p* < 0.05; **: *p* < 0.01; ***: *p* < 0.001; ****: *p* < 0.0001; ns: *p* > 0.05).

**Table 2:** Rotarod test values. The table represents the mean time in seconds (s) and the standard deviation for the time that untreated (vehicle) or treated (folinic acid, FA) animals remained walking on the rod. The *p* value indicates the statistical significance between the vehicle group and the FA-treated group (ns: *p* > 0.05).

Day	Vehicle (s)	FA (s)	*p* Value
−1	158.5 ± 5.32	159.6 ± 8.26	ns
1	12.67 ± 4.04	21.4 ± 3.44	ns
2	12.17 ± 4.95	21 ± 3.08	ns
3	19.5 ± 5.28	33 ± 2.73	*p* < 0.01
4	25.17 ± 5.30	47 ± 7.03	*p* < 0.0001
5	46.83 ± 5.84	57 ± 6.36	*p* < 0.05
6	69.17 ± 4.96	82.4 ± 10.69	*p* < 0.01
7	84.83 ± 10.52	100.2 ± 10.52	*p* < 0.01
10	113 ± 15.59	123 ± 4.85	*p* < 0.05
14	119.17 ± 9.75	157.4 ± 11.24	*p* < 0.0001

## Data Availability

Data is contained within the article.
